# Supplementary figures and images for: Waterlogging tolerance and recovery capability screening in peanut: a comparative analysis of waterlogging effects on physiological traits and yield
Source: PeerJ. 2022 Jan 12;10:e12741. doi: 10.7717/peerj.12741 (PMC8760856; doi:10.7717/peerj.12741)

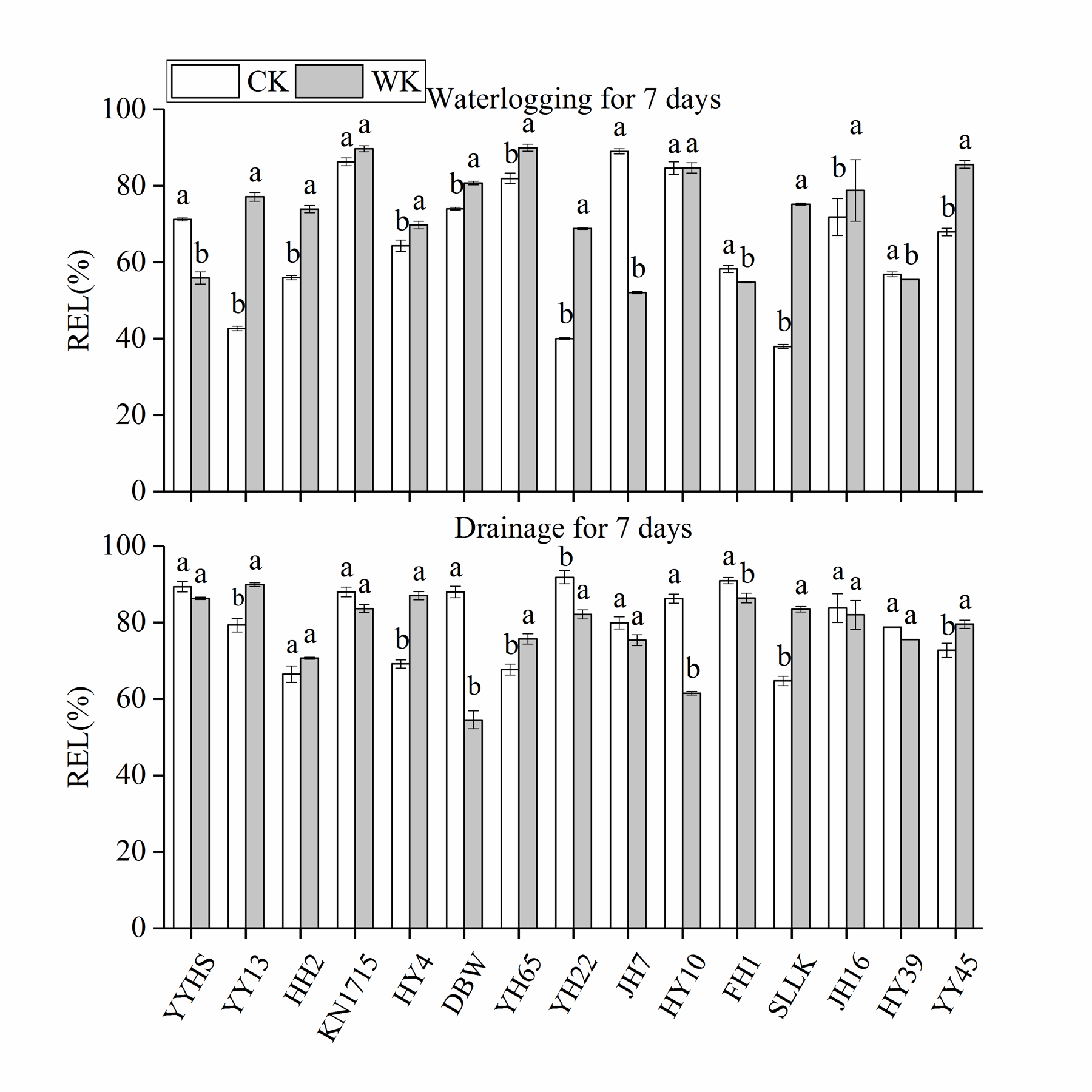

Supplement: Supplemental Information 2 — Data represents the mean ± standard error. Letters a and b represent statistically significant differences (p < 0.05 within a variety under control treatment (CK) and waterlogging treatment (WK) as determined by the least significant difference test. [file peerj-10-12741-s002.png]

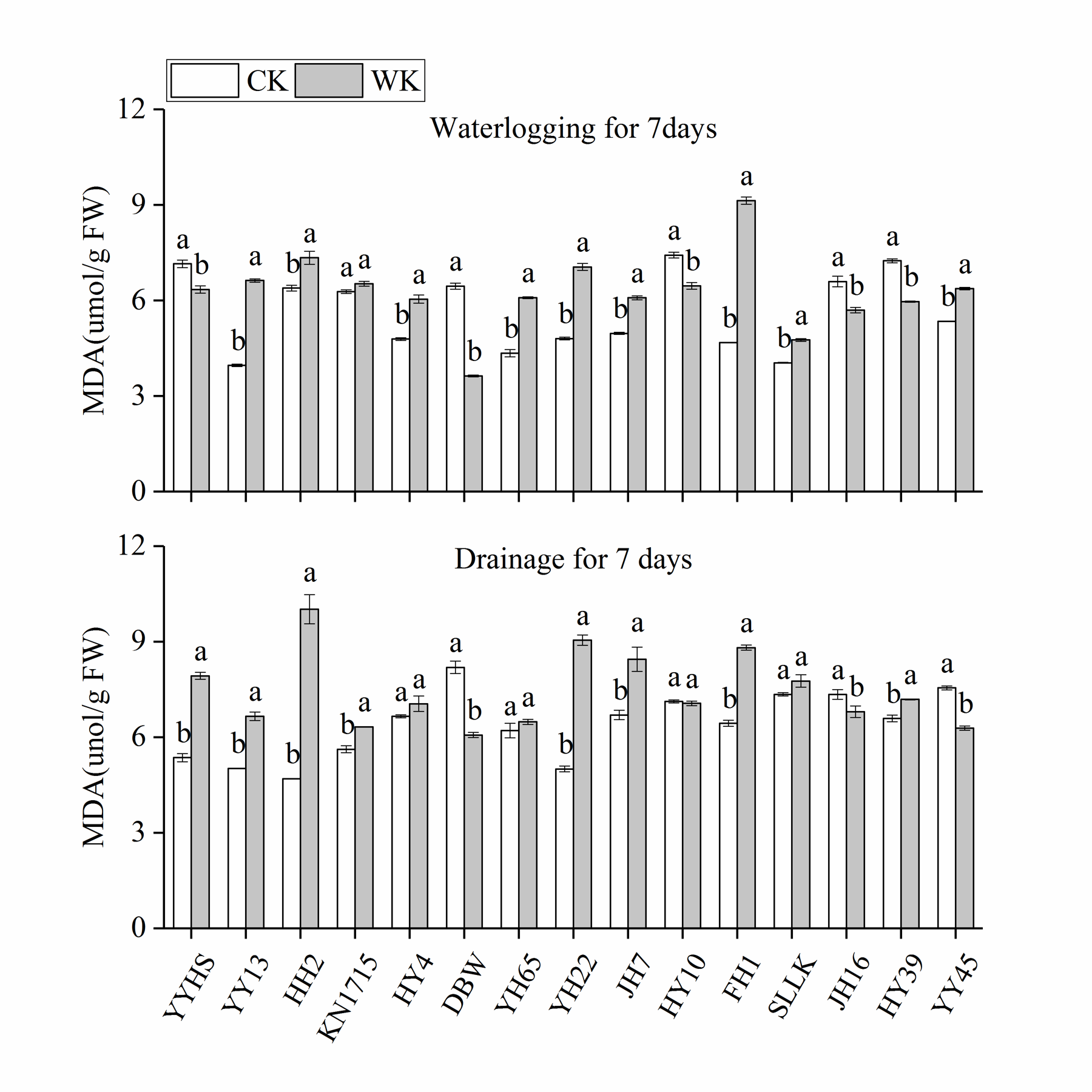

Supplement: Supplemental Information 3 — Data represents the mean ± standard error. Letters a and b represent statistically significant differences (p < 0.05) within a variety under control treatment (CK) and waterlogging treatment (WK) as determined by the least significant difference test. [file peerj-10-12741-s003.png]

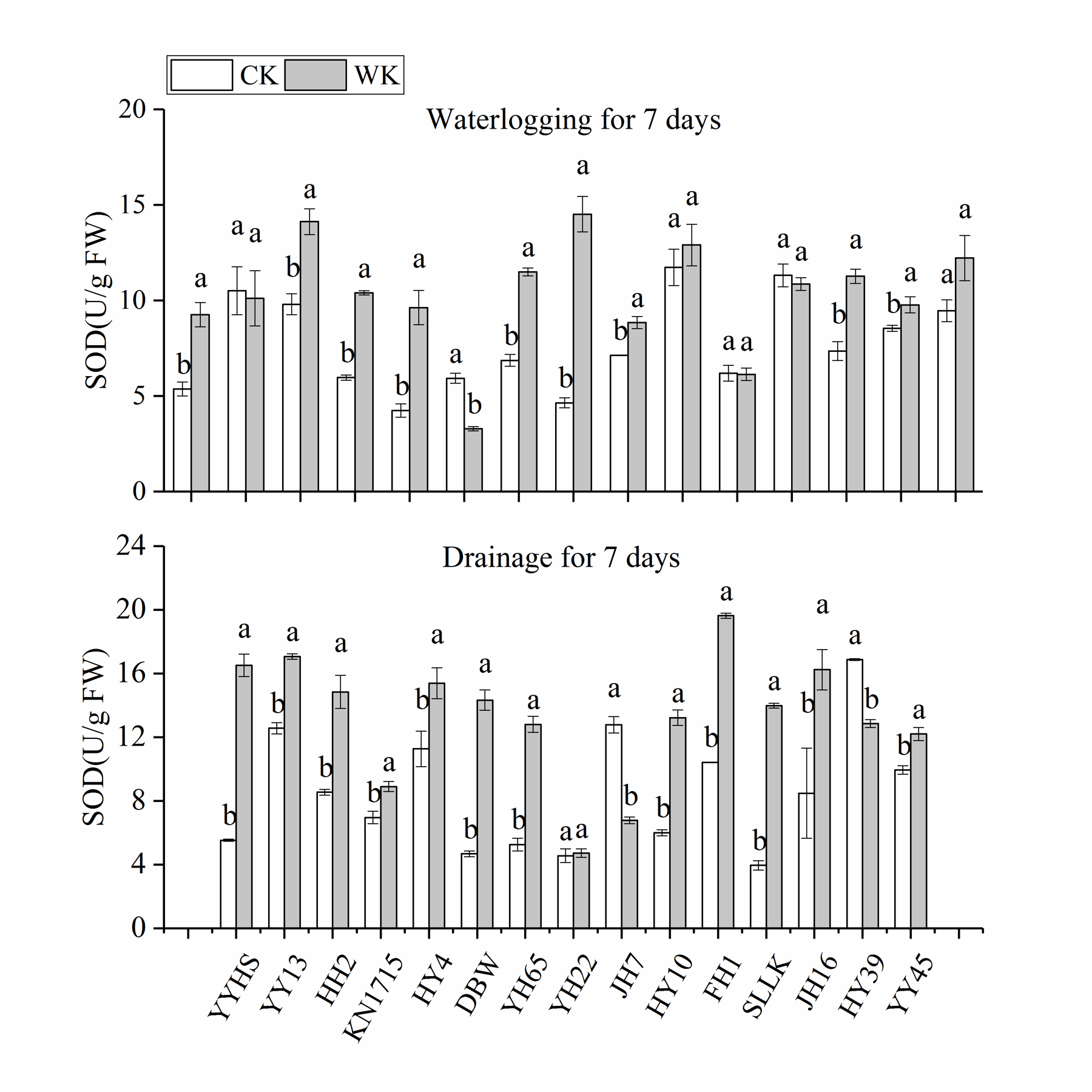

Supplement: Supplemental Information 4 — Data represents the mean ± standard error. Letters a and b represent statistically significant differences (p < 0.05) within a variety under control treatment (CK) and waterlogging treatment (WK) as determined by the least significant difference test. [file peerj-10-12741-s004.png]

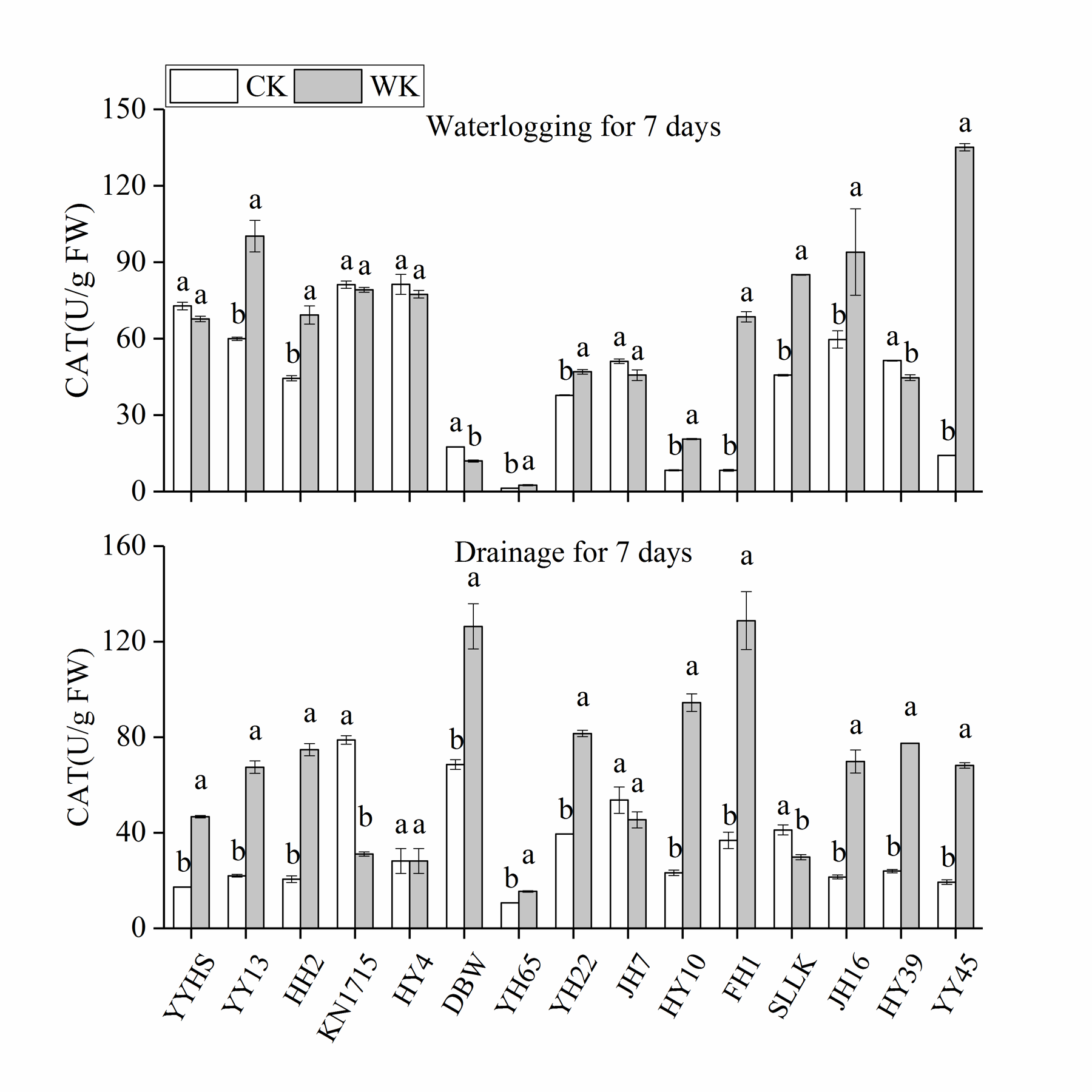

Supplement: Supplemental Information 5 — Data represents the mean ± standard error. Letters a and b represent statistically significant differences (p < 0.05) within a variety under control treatment (CK) and waterlogging treatment (WK) as determined by the least significant difference test. [file peerj-10-12741-s005.png]

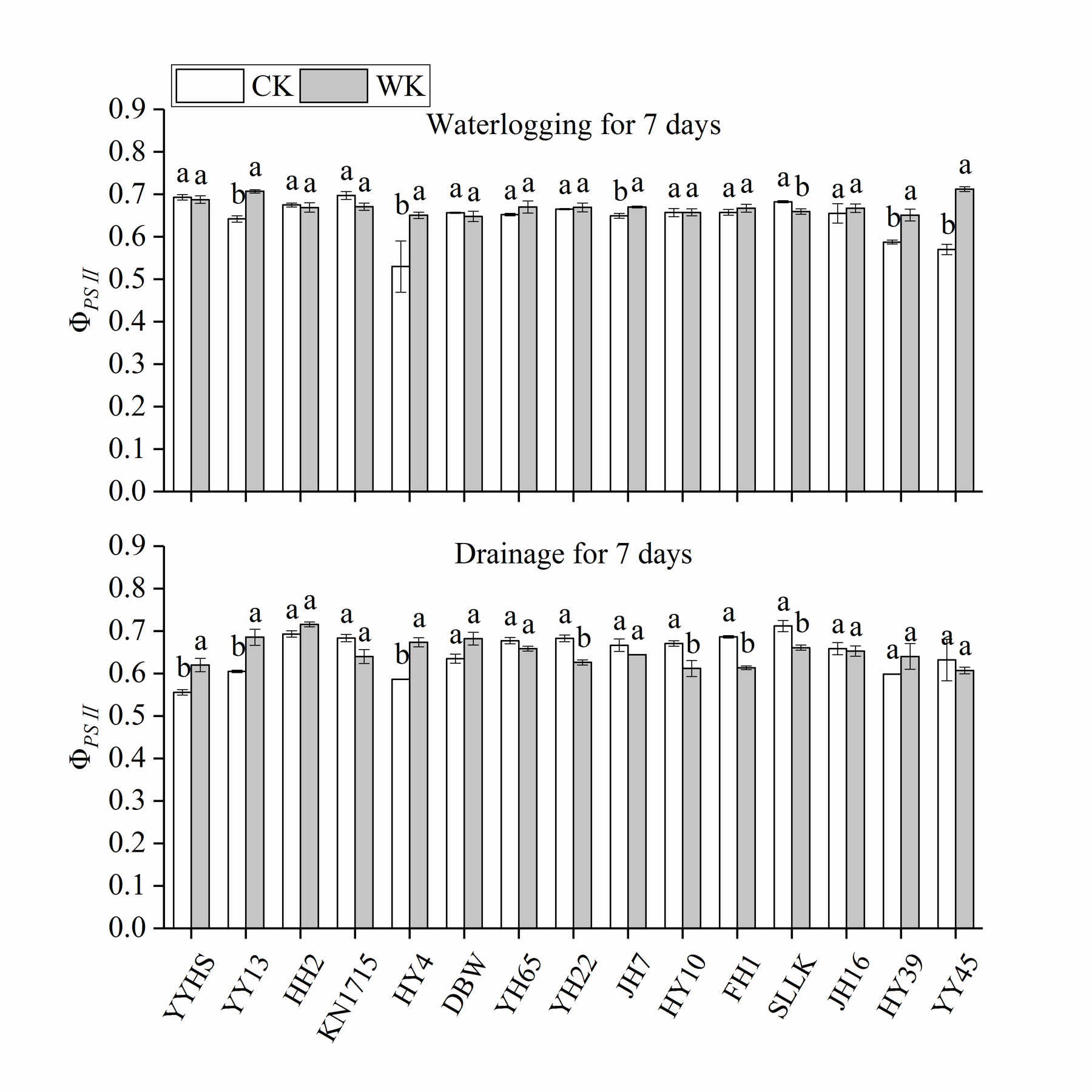

Supplement: Supplemental Information 6 — Data represents the mean ± standard error. Letters a and b represent statistically significant differences (p < 0.05) within a variety under control treatment (CK) and waterlogging treatment (WK) as determined by the least significant difference test. [file peerj-10-12741-s006.png]

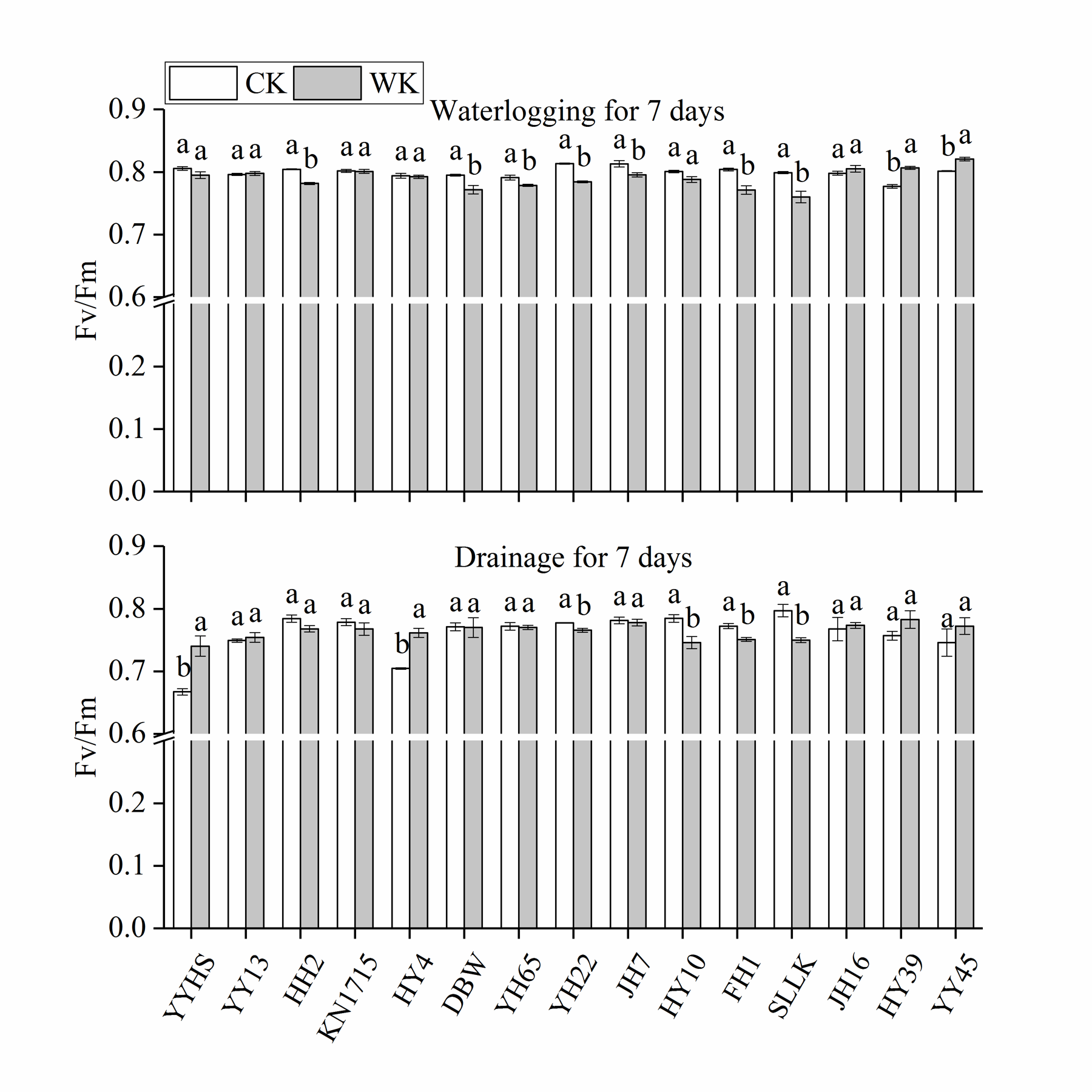

Supplement: Supplemental Information 7 — Data represents the mean ± standard error. Letters a and b represent statistically significant differences (p < 0.05) within a variety under control treatment (CK) and waterlogging treatment (WK) as determined by the least significant difference test. [file peerj-10-12741-s007.png]

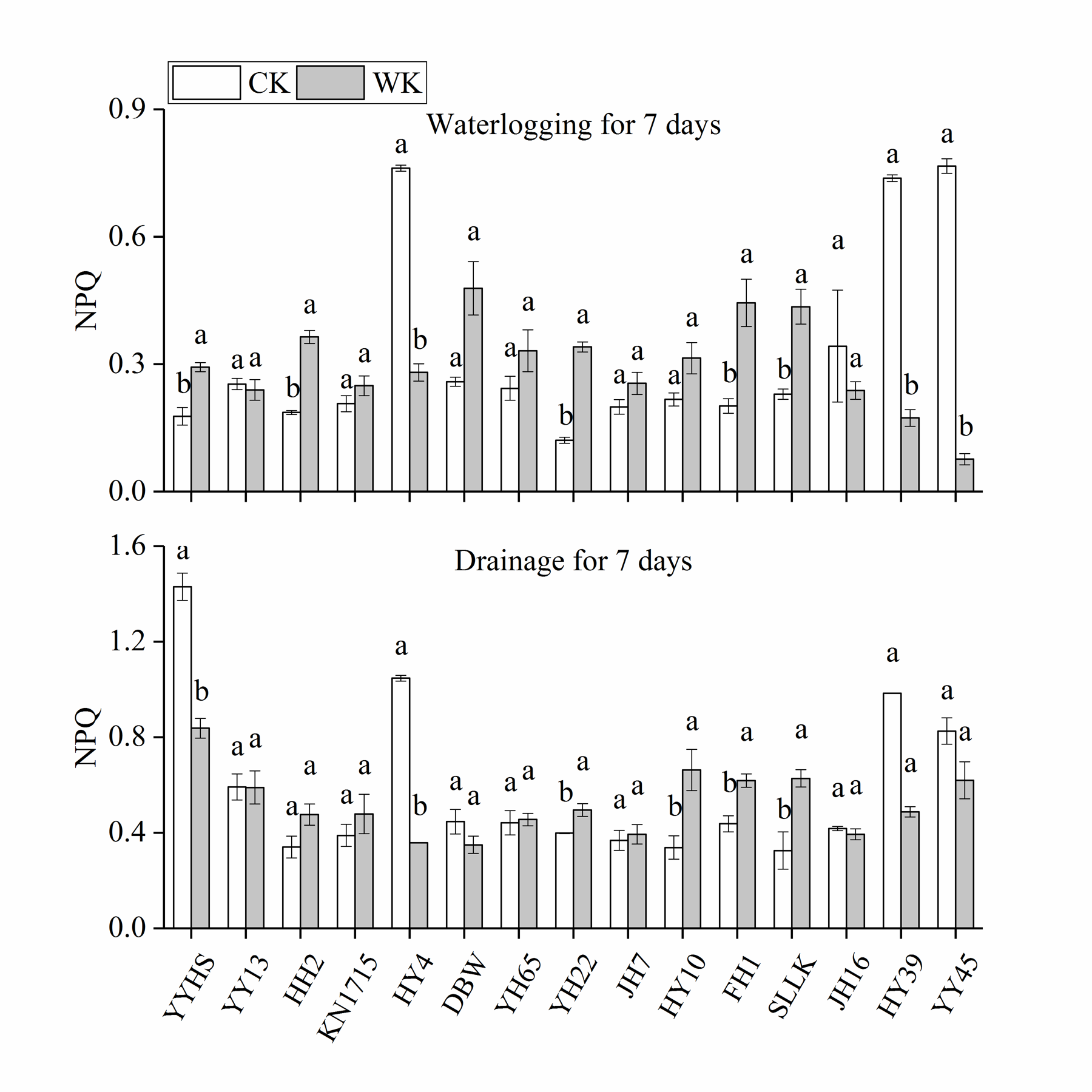

Supplement: Supplemental Information 8 — Data represents the mean ± standard error. Letters a and b represent statistically significant differences (p < 0.05) within a variety under control treatment (CK) and waterlogging treatment (WK) as determined by the least significant difference test. [file peerj-10-12741-s008.png]

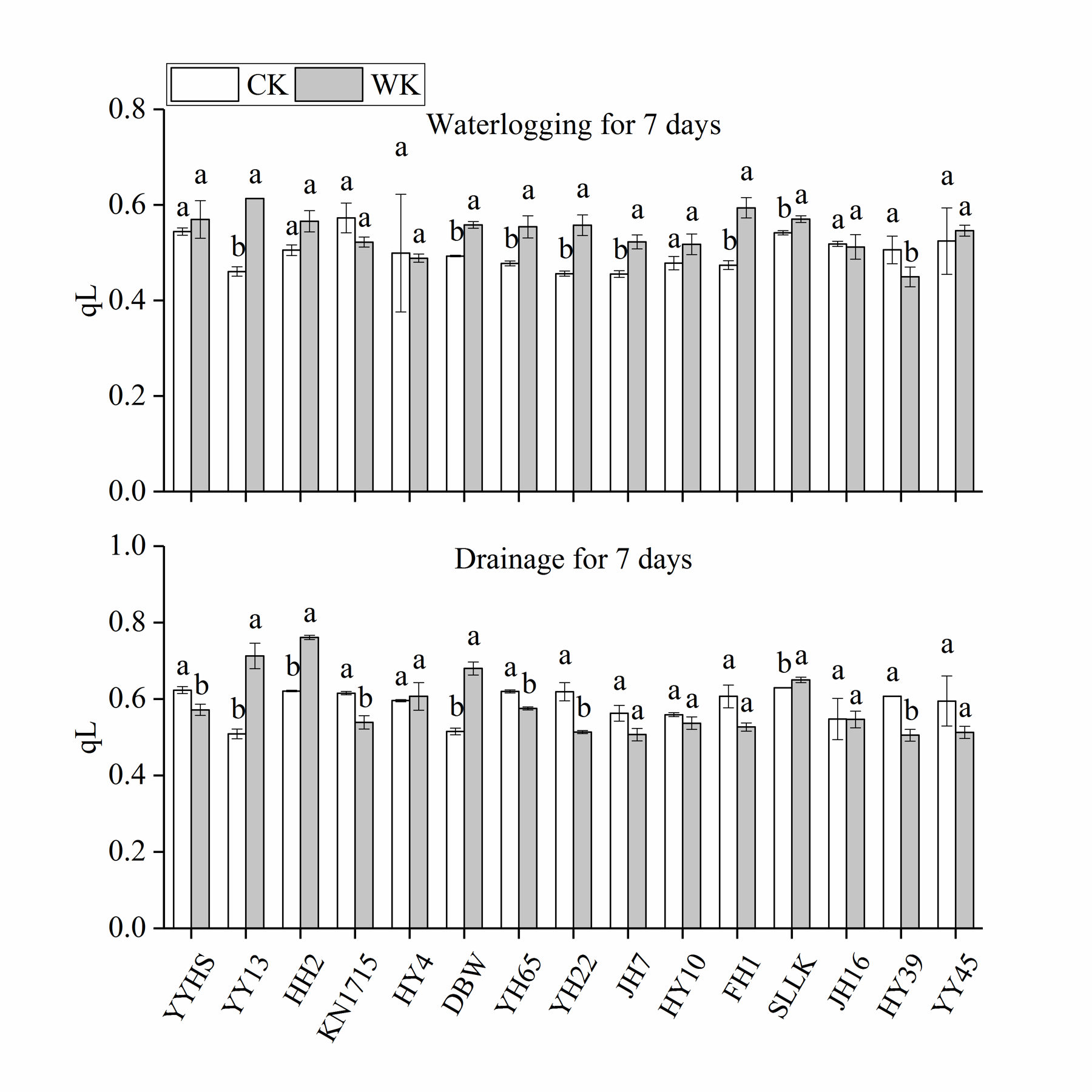

Supplement: Supplemental Information 9 — Data represents the mean ± standard error. Letters a and b represent statistically significant differences (p < 0.05) within a variety under control treatment (CK) and waterlogging treatment (WK) as determined by the least significant difference test. [file peerj-10-12741-s009.png]

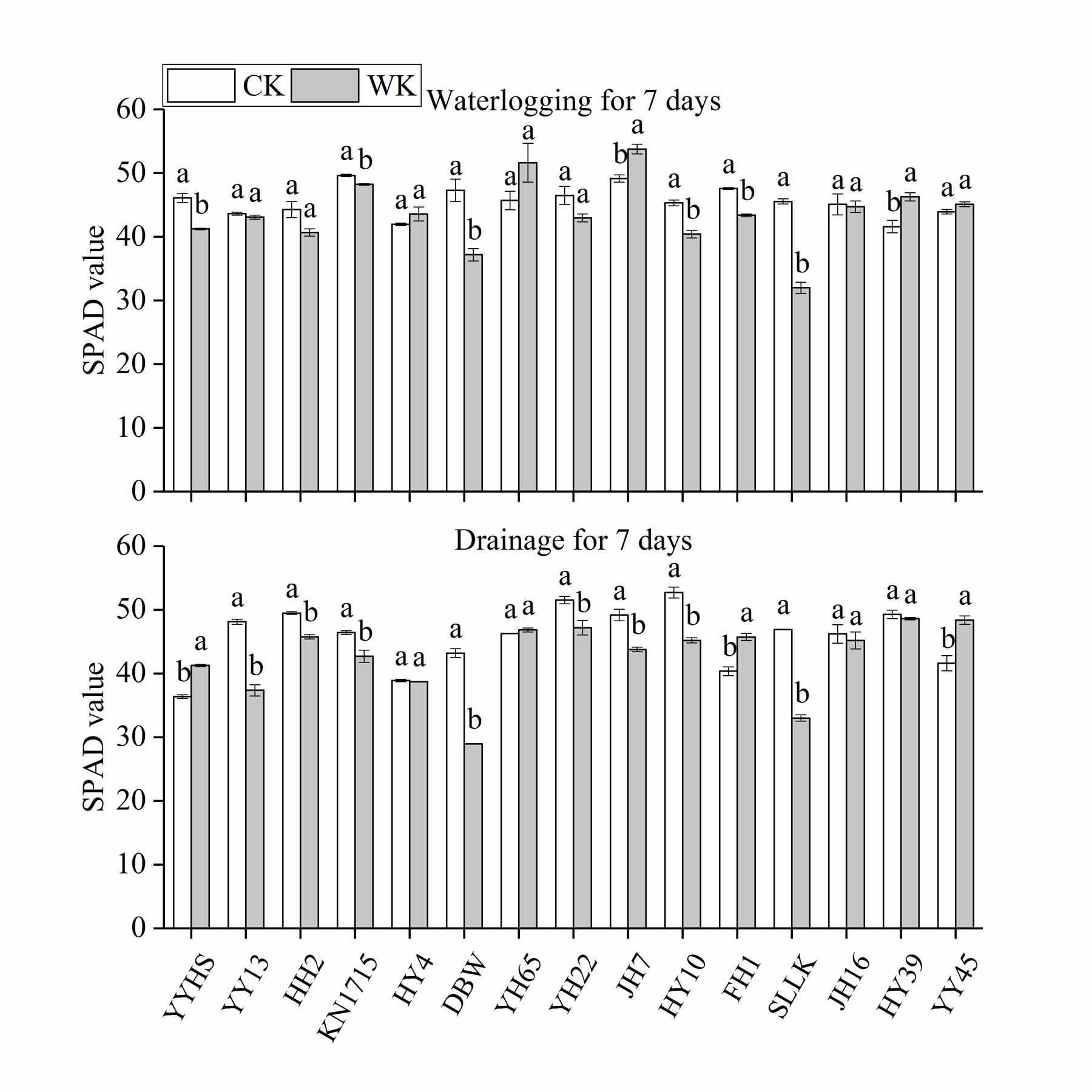

Supplement: Supplemental Information 10 — Data represents the mean ± standard error. Letters a and b represent statistically significant differences (p < 0.05) within a variety under control treatment (CK) and waterlogging treatment (WK) as determined by the least significant difference test. [file peerj-10-12741-s010.png]

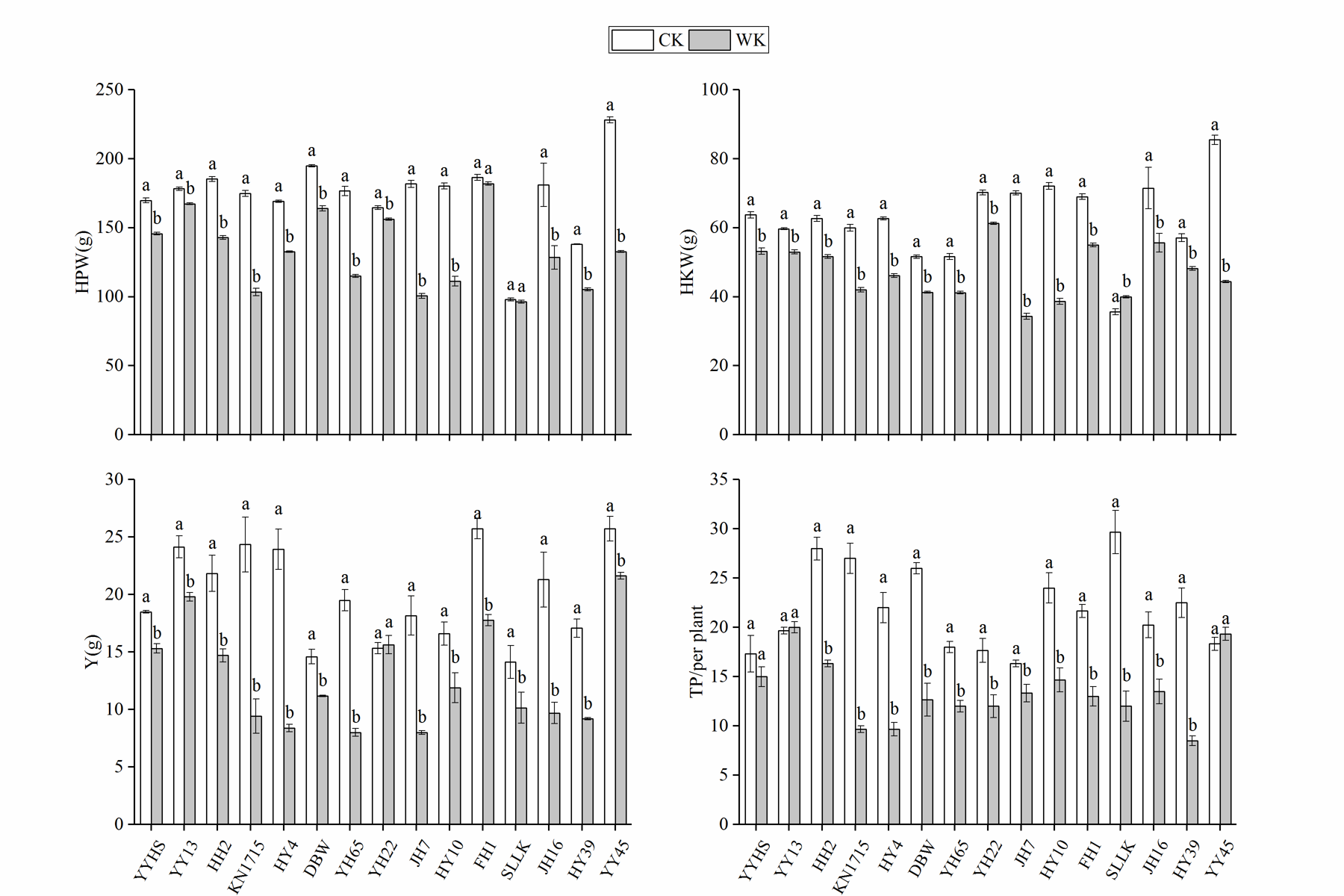

Supplement: Supplemental Information 11 — Data represents the mean ± standard error. Letters a and b represent statistically significant differences (p < 0.05) within a variety under control treatment (CK) and waterlogging treatment (WK) as determined by the least significant difference test. HPW: hundred pods weight; HKW: hundred kernels weight; Y: yield per hectare; TP: the number of total pods per plant. [file peerj-10-12741-s011.png]
